# BRCA Challenge: BRCA Exchange as a global resource for variants in *BRCA1* and *BRCA2*

**DOI:** 10.1371/journal.pgen.1007752

**Published:** 2018-12-26

**Authors:** Melissa S. Cline, Rachel G. Liao, Michael T. Parsons, Benedict Paten, Faisal Alquaddoomi, Antonis Antoniou, Samantha Baxter, Larry Brody, Robert Cook-Deegan, Amy Coffin, Fergus J. Couch, Brian Craft, Robert Currie, Chloe C. Dlott, Lena Dolman, Johan T. den Dunnen, Stephanie O. M. Dyke, Susan M. Domchek, Douglas Easton, Zachary Fischmann, William D. Foulkes, Judy Garber, David Goldgar, Mary J. Goldman, Peter Goodhand, Steven Harrison, David Haussler, Kazuto Kato, Bartha Knoppers, Charles Markello, Robert Nussbaum, Kenneth Offit, Sharon E. Plon, Jem Rashbass, Heidi L. Rehm, Mark Robson, Wendy S. Rubinstein, Dominique Stoppa-Lyonnet, Sean Tavtigian, Adrian Thorogood, Can Zhang, Marc Zimmermann, BRCA Challenge Authors, John Burn, Stephen Chanock, Gunnar Rätsch, Amanda B. Spurdle

**Affiliations:** 1 University of California Santa Cruz Genomics Institute, University of California, Santa Cruz, California, United States of America; 2 Broad Institute, Cambridge, Massachusetts, United States of America; 3 Genetics and Computational Biology Division, QIMR Berghofer Medical Research Institute, Brisbane, Queensland, Australia; 4 Department of Biomolecular Engineering, University of California Santa Cruz, Santa Cruz, California, United States of America; 5 Department of Computer Science, Biomedical Informatics Group Universitätsstrasse, Zürich, Switzerland; 6 Biomedical Informatics, University Hospital Zurich, Zurich, Switzerland; 7 Biocybernetics Laboratory, Computer Science Department, University of California, Los Angeles, California, United States of America; 8 Centre for Cancer Genetic Epidemiology, Department of Public Health and Primary Care, University of Cambridge, Cambridge, United Kingdom; 9 Medical Genomics and Metabolic Genetics Branch, National Human Genome Research Institute, Bethesda, Maryland, United States of America; 10 School for the Future of Innovation in Society, and Consortium for Science, Policy & Outcomes, Arizona State University, Tempe, Arizona, United States of America; 11 Department of Health Sciences Research, Mayo Clinic, Rochester, Minnesota, United States of America; 12 The Global Alliance for Genomics and Health, Toronto, Ontario, Canada; 13 Department of Human Genetics, Leiden University Medical Center, Leiden, The Netherlands; 14 Centre of Genomics and Policy, Faculty of Medicine, McGill University, Montreal, Quebec, Canada; 15 Perelman School of Medicine at the University of Pennsylvania, Philadelphia, Pennsylvania, United States of America; 16 Program in Cancer Genetics, Department of Oncology and Human Genetics, McGill University, Montréal, Quebec, Canada; 17 Dana-Farber Cancer Institute, Harvard Medical School, Boston, Massachusetts, United States of America; 18 Huntsman Cancer Institute and Department of Dermatology, University of Utah, Salt Lake City, Utah, United States of America; 19 Partners HealthCare Laboratory for Molecular Medicine and Harvard Medical School, Boston, Massachusetts, United States of America; 20 Graduate School of Medicine, Osaka University, Osaka, Japan; 21 Centre of Genomics and Policy, Faculty of Medicine, Human Genetics, McGill University, Montreal, Québec, Canada; 22 Center for Biomolecular Science & Engineering, University of California, Santa Cruz, California, United States of America; 23 Invitae, San Francisco, California, United States of America; 24 Clinical Genetics Service, Department of Medicine, Memorial Sloan Kettering Cancer Center, New York, New York, United States of America; 25 Department of Pediatrics, Baylor College of Medicine, Houston, Texas, United States of America; 26 Department of Molecular and Human Genetics, Baylor College of Medicine, Houston, Texas, United States of America; 27 National Disease Registration, National Cancer Registration and Analysis Service, Public Health England, London, United Kingdom; 28 Laboratory for Molecular Medicine, Partners Healthcare Personalized Medicine, Cambridge, Massachusetts, United States of America; 29 Department of Pathology, Brigham & Women's Hospital and Harvard Medical School, Boston, Massachusetts, United States of America; 30 CancerLinQ at American Society of Clinical Oncology (ASCO), Alexandria, Virginia, United States of America; 31 Institut Curie, Cancer Genetic Clinic, Paris, France; 32 Department of Oncological Sciences, The University of Utah, Salt Lake City, Utah, United States of America; 33 Centre of Genomics and Policy, McGill University, Montreal, Canada; 34 Department of Computer Science, University of California, Santa Cruz, Santa Cruz, California, United States of America; 35 Institute of Genetic Medicine, Newcastle University, Centre for Life, Newcastle upon Tyne, United Kingdom; 36 Division of Cancer Epidemiology and Genetics, National Cancer Institute, Rockville, Maryland, United States of America; 37 Computational Biology Program, Memorial Sloan Kettering Cancer Center, New York, New York, United States of America; 38 Swiss Institute for Bioinformatics, Lausanne, Switzerland; 39 Genetics and Computational Biology Division, QIMR Berghofer Medical Research Institute, Herston, Brisbane, Australia; Cleveland Clinic Genomic Medicine Institute, UNITED STATES

## Abstract

The BRCA Challenge is a long-term data-sharing project initiated within the Global Alliance for Genomics and Health (GA4GH) to aggregate *BRCA1* and *BRCA2* data to support highly collaborative research activities. Its goal is to generate an informed and current understanding of the impact of genetic variation on cancer risk across the iconic cancer predisposition genes, *BRCA1* and *BRCA2*. Initially, reported variants in *BRCA1* and *BRCA2* available from public databases were integrated into a single, newly created site, www.brcaexchange.org. The purpose of the BRCA Exchange is to provide the community with a reliable and easily accessible record of variants interpreted for a high-penetrance phenotype. More than 20,000 variants have been aggregated, three times the number found in the next-largest public database at the project’s outset, of which approximately 7,250 have expert classifications. The data set is based on shared information from existing clinical databases—Breast Cancer Information Core (BIC), ClinVar, and the Leiden Open Variation Database (LOVD)—as well as population databases, all linked to a single point of access. The BRCA Challenge has brought together the existing international Evidence-based Network for the Interpretation of Germline Mutant Alleles (ENIGMA) consortium expert panel, along with expert clinicians, diagnosticians, researchers, and database providers, all with a common goal of advancing our understanding of *BRCA1* and *BRCA2* variation. Ongoing work includes direct contact with national centers with access to *BRCA1* and *BRCA2* diagnostic data to encourage data sharing, development of methods suitable for extraction of genetic variation at the level of individual laboratory reports, and engagement with participant communities to enable a more comprehensive understanding of the clinical significance of genetic variation in *BRCA1* and *BRCA2*.

## Author summary

The goal of this study and paper has been to develop an international resource to generate an informed and current understanding of the impact of genetic variation on cancer risk across the cancer predisposition genes, *BRCA1* and *BRCA2*. Reported variants in *BRCA1* and *BRCA2* available from public databases were integrated into a single, newly created site, www.brcaexchange.org, to provide a reliable and easily accessible record of variants interpreted for a high-penetrance phenotype.

## Introduction

Over 60 years ago, geneticists began to correlate cytogenetic observations with clinical significance. Over time, a succession of new genomic technologies has enabled investigators to interrogate single-base sequence analyses in clinically informative settings. This led to the emergence of molecular diagnostics, which, in turn, prompted the development of variant databases curated by academic and/or national groups. McKusick’s classic textbook evolved into Online Mendelian Inheritance in Man (OMIM; https://omim.org/), whereas a number of international efforts were launched to standardize nomenclature and classification—such as the Human Genome Organization (http://www.hugo-international.org/), Orphanet (www.orpha.net), the Human Genome Variation Society (HGVS; www.hgvs.org), and the Human Variome Project (HVP)[1]—as well as reference databases, including the Human Gene Mutation Database (HGMD) (http://www.hgmd.cf.ac.uk/), DatabasE of genomiC varIation and Phenotype in Humans using Ensembl Resources (DECIPHER; https://decipher.sanger.ac.uk/), Leiden Open Variation Database (LOVD; www.lovd.nl), and ClinVar (https://www.ncbi.nlm.nih.gov/clinvar/). Over the last decade, the expansion of next-generation sequencing and its deployment in routine diagnosis have made variant interpretation a critical rate-limiting factor in the realization of clinical benefit.

At the first partner meeting of the Global Alliance for Genomics and Health (GA4GH) in 2014, it was agreed to focus one of its demonstration projects on the problem of variant capture and interpretation, drawing on both traditional clinical resources and the availability of “big data” to illuminate normal variation. The genes *BRCA1* and *BRCA2* were chosen because of their iconic status in the public consciousness and the scale of routine use of genetic test results to guide major clinical decisions. At the recent launch of the restructured GA4GH, “GA4GH Connect” [2], BRCA Challenge was identified as one of the inaugural driver projects for an international effort to enable more robust genomic data sharing in the future. As of October 2017, it has been relaunched as an independent effort and is working with GA4GH to engage in the strategic development and uptake of data sharing frameworks and standards. From the outset, the BRCA Challenge has convened a spectrum of stakeholders to accurately interpret *BRCA1* and *BRCA2* variation, to make this information freely available worldwide, and to improve the care of patients who present with variants in *BRCA1* or *BRCA2* (Box 1).

**Box 1. Specific goals of the BRCA Challenge**

1. Share *BRCA1* and *BRCA2* variants publicly.
2. Create an online environment for collaborative variant curation with access to evidence (e.g., phenotypes, family history, genetic data, and functional studies).
3. Create a curated list of *BRCA1* and *BRCA2* variants, interpreted by expert consensus, to serve as a reference for accurate clinical care based on criteria established by the ENIGMA consortium.
4. Address social, ethical, and legal challenges to global data sharing, and engage with patient advocacy organizations from around the world.
5. Create a model system for data sharing for other disease genes.

Inherited variation in the *BRCA1* and *BRCA2* genes can indicate genetic predisposition to breast, ovarian, and other cancers, but a large proportion of observed variants are not disease associated ("pathogenic")[3, 4]. In response to the need for an international solution to provide a comprehensive annotation of *BRCA1* and *BRCA2* variants, we have developed the BRCA Exchange—a single comprehensive portal to display global *BRCA* variation, linking current structures and resources while encouraging deposition of new data. The BRCA Exchange is based on shared data from clinicians, clinical laboratories, and researchers across the world, including existing clinical databases—Breast Cancer Information Core (BIC), ClinVar, and LOVD—linked to a single point of access. An international interpretation community includes the Evidence-based Network for the Interpretation of Germline Mutant Alleles (ENIGMA) consortium [5], established to investigate the clinical significance of variants in breast−ovarian cancer predisposition genes, and which is recognized by Clinical Genome Resource (ClinGen) as an expert panel for interpretation of *BRCA1* and *BRCA2* variants. The purpose of the Exchange is to provide the community with a reliable and easily accessible record of variants interpreted as pathogenic for a high penetrance phenotype; the Exchange also reports on variants deemed “not clinically relevant” (nonpathogenic) and eventually will report on reduced penetrance variants associated with a moderate or modest risk for a spectrum of cancers (such as *BRCA2* p.K3326*)[6].

### The challenge of interpreting *BRCA1* and *BRCA2* variants

In 1866, the French physician Paul Broca described a cluster of breast cancers in his wife’s family, heralding the concept of familial risk for breast cancer [7]. The concept of a cancer-predisposition gene emerged as a critical driving force for applying the evolving tools of genetic analyses to map cancer genes in families presenting with breast and/or ovarian cancer; this view emerged over decades of studies describing familial clusters, twin studies, and special populations at high risk for breast and ovarian cancer. In 1990, what is now known as *BRCA1* was localized to the long arm of chromosome 17 through analyses of high-risk families [8]. Four years later, positional cloning and mutational analyses identified the gene itself and revealed specific coding variants (predominantly truncating alleles) strongly linked to risk for breast and ovarian cancers within families and high-risk populations [9]. A second major cancer gene for breast and ovarian cancer, *BRCA2*, was localized to the long arm of chromosome 13 in 1994 and cloned and sequenced in 1995 [10–12]. By 1996, genetic testing of these two genes was introduced into clinical practice. Although millions of women, and more recently men, at risk for cancer have been tested for germline genetic variants in *BRCA1* and *BRCA2*, diagnostic sequencing has continued to identify variants with uncertain relevance to cancer risk. This has emerged as a particularly challenging issue for determination of risk for not only breast and ovarian cancer but now aggressive prostate and pancreatic cancers. What has emerged is that whereas some germline variants confer high risk for cancer due to disruption of gene function [13, 14], many *BRCA1* and *BRCA2* variants identified during routine genetic testing are determined to have little or no clinical significance with respect to cancer risk [4, 15, 16].

Despite the low overall prior probability of pathogenicity for a variant of uncertain significance in these two genes [4, 17], such variants nevertheless present a clinical challenge in that they complicate test reporting and medical decision-making for clinicians and patients, and carriers of “equivocal” variants are more likely to over-interpret their result, seek additional management, or in some circumstances, receive overtreatment by clinicians outside clinical genetics [18–20]. There are also *BRCA1* and *BRCA2* variants that appear to be associated with a modest or moderate (but still elevated) risk of breast and ovarian cancer, compared with “traditional” deleterious mutations. The clinical reporting on these variants is often inconsistent and therefore confusing [6, 21–24].

Over decades of study and sequencing at-risk individuals for these two genes and others, it has become clear that there is a need for a consistent variant classification scheme that is systematically developed, validated, peer-reviewed, and published as authority for use by diverse stakeholders. Previously, the International Agency for Cancer Research (IARC) [25] and the American College of Medical Genetics and Genomics (ACMG) with the Association for Molecular Pathology (AMP) [26] proposed schemes for classification of variants as pathogenic or nonpathogenic, incorporating evidence using quantitative and qualitative approaches. The utility of these classification efforts has been seen in their incorporation into clinical pipelines, but the practice of interpreting variants remains a daunting challenge. It may be difficult for individual testing laboratories to have sufficient clinical or laboratory information to assign classification for rarely encountered variants. Furthermore, the rapid increase in sequence-based testing, particularly among individuals with no personal or family history of cancer, has led to more complex variant interpretation. Many sequencing centers have begun to aggregate observed variants in databases and to classify them using clinical information and variant-level evidence from multiple sources—thereby reducing uncertainty. However, even between large laboratories, discordant reporting of variant interpretations continues due to the balkanization of evidence and differences between curators in utilization of “standard” classification criteria and/or publicly available evidence [27].

The ENIGMA consortium (http://www.enigmaconsortium.org) is an international multidisciplinary consortium [5], currently comprising more than 300 listed members from over 190 research groups and/or clinical testing laboratories spanning more than 35 countries. The aim of the ENIGMA consortium is to develop methods for improved classification of variants in *BRCA1* and *BRCA2* and other known or suspected breast cancer predisposition genes to guide their use in clinical testing panels. The National Institutes of Health (NIH)-funded ClinGen (https://www.clinicalgenome.org/) has a process to appoint expert panels to provide expert variant curation for specific genes. The ENIGMA consortium was approved as an expert panel by ClinGen to apply its methods to define more precise criteria for annotation of variants in *BRCA1* and *BRCA2*.

It is anticipated that the number of variants of uncertain clinical significance (VUSs) and differences in interpretation of the same variant between different laboratories should decrease with time as public databases approach a fully comprehensive annotation of variants in familial and population-based settings together with availability of the associated information relevant to variant classification. It is the express purpose of the BRCA Challenge project and the BRCA Exchange website to accelerate this process, using *BRCA1* and *BRCA2* as demonstration genes. In this regard, interpretation of currently identified VUSs can be accelerated by enabling increased sharing of existing variant information and using the additional observations to improve evidence-based classification. Unfortunately, the willingness to share varies among data holders for a variety of reasons, including ethical and legal challenges with respect to sharing patient information, resource limitations, and commercial and national health system models commodifying patient and variant data. Databases often aggregate incompletely curated variant classifications with little or no supporting evidence, further muddying the waters of appropriate interpretation and clinical care.

To date, the scope of *BRCA1* and *BRCA2* testing has been substantially larger than any other cancer predisposition gene, yet the ability to look across the spectrum of publicly available data has not been coordinated. The emergence of multiple public and private databases worldwide, together with proprietary diagnostic testing databases, have retarded efforts to link a large set of well-annotated variants for public interpretation. The rapid rise of next-generation sequencing by academic centers, commercial diagnostic companies, and national programs has created an additional opportunity to collect information—especially variants derived from non-*BRCA1* and *BRCA2* familial cases—and use these to classify more variants. Together, these factors underscore how *BRCA1* and *BRCA2* are a compelling case for sharing data to advance the precision of variant interpretation, thus enabling clinicians and patients to make important decisions together.

### Development of a public portal for *BRCA1* and *BRCA2* variants: BRCA Exchange

**Fig 1:**
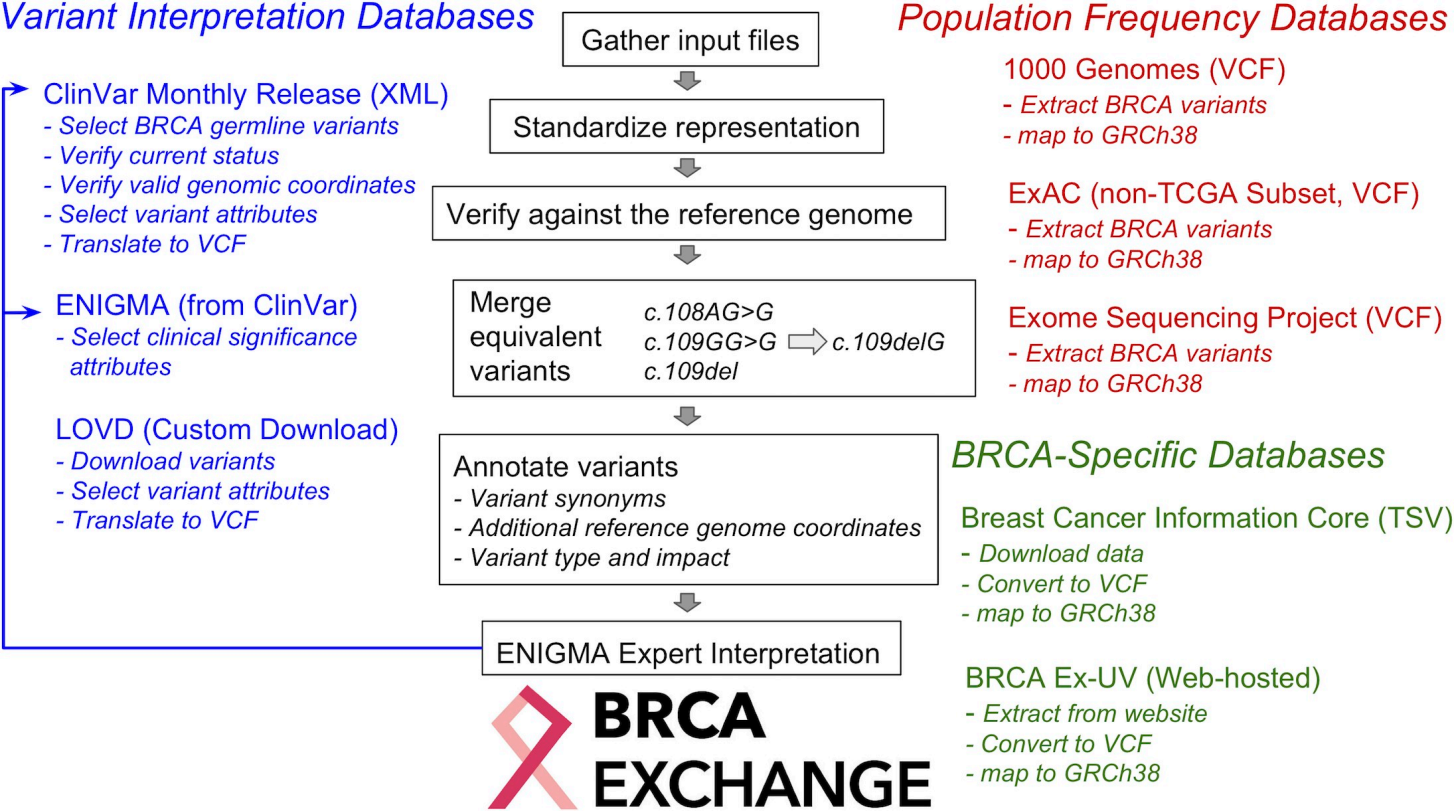
Data flow in the BRCA Exchange pipeline. BRCA Exchange combines information from major public sources to offer a comprehensive view of BRCA variants from a single web portal. It combines variant information from ClinVar, ENIGMA (as the ClinGen expert panel on BRCA variation) and LOVD (blue); population frequency data from 1000 Genomes, ExAC, and the ESP (red); and BRCA-specific information from the BIC on BRCA 1 and 2 Ex-UV (green). Each month, BRCA Exchange collects variant data from these sources and translates them into a consistent representation. It verifies that all variants are consistent in the reference bases with the reference human genome and discards any variant data that are inconsistent with the genome. Next, it identifies functionally equivalent variants, in which two or more variants might produce the same alternative allele despite distinct representation, and merges equivalencies to generate a set of distinct BRCA variants. It gathers annotations, such as functional impact terms and alternative variant names. Finally, it compares the new variant data to the previous month to identify any variants that are new or updated. These data are shared publicly at brcaexchange.org. The ENIGMA consortium analyzes these aggregated data to determine the clinical significance of unreviewed variants and deposits these interpretations in ClinVar, completing the cycle of information. BIC, Breast Cancer Information Core; ENIGMA, Evidence-based Network for the Interpretation of Germline Mutant Alleles; ESP, Exome Sequencing Project; ExAC, Exome Aggregation Consortium; BRCA Ex-UV, BRCA1 and BRCA2 Ex-UV; GRCh38, Genome Reference Consortium Human Build 38 Organism; LOVD, Leiden Open Variation Database; TCGA, The Cancer Genome Atlas; TSV, Tab Separated Values; VCF, Variant Format Call; XML, Extensible Markup Language.

The BRCA Challenge has developed an open-access web portal, the BRCA Exchange (http://brcaexchange.org), a resource to display *BRCA1* and *BRCA2* variants drawn from global sources and to enable *BRCA1* and *BRCA2* variants to be expert-reviewed, interpreted, classified, and aggregated in an integrated data system (Fig 1). The publicly accessible display of these classifications, with supporting evidence, facilitates accurate understanding of the clinical relevance of any individual *BRCA1* or *BRCA2* variant. The portal is fully open access and enables easy download of variants and variant-level evidence by anyone with an internet connection. The global focus of the portal enables coordination and collaboration with relevant investigators and databases from around the world in a supranational consortium, thus creating the most comprehensive source of variation in *BRCA1* and *BRCA2*, including high-quality variant classifications made by ENIGMA.

To ensure broad usage of the BRCA Exchange, features have been added to maximize utility of the data. The default view displays only expert-curated variant classifications, in order to support layperson engagement with the data. Users who wish to access more extensive data can click through a disclaimer and arrive at a version of the portal that displays the full database, inclusive of classifications made by LOVD and ClinVar submitters and curators other than ENIGMA, with potential interpretation conflicts. Data quality is ensured by the back-end pipeline, which merges equivalent variants and filters out variants with erroneous nomenclature terms or reference bases that are inconsistent with the current reference genome. These data can be downloaded in full and are available for third-party use. The Exchange data are also versioned with each new data upload, in line with regulations for clinical-grade databases.

**Fig 2:**
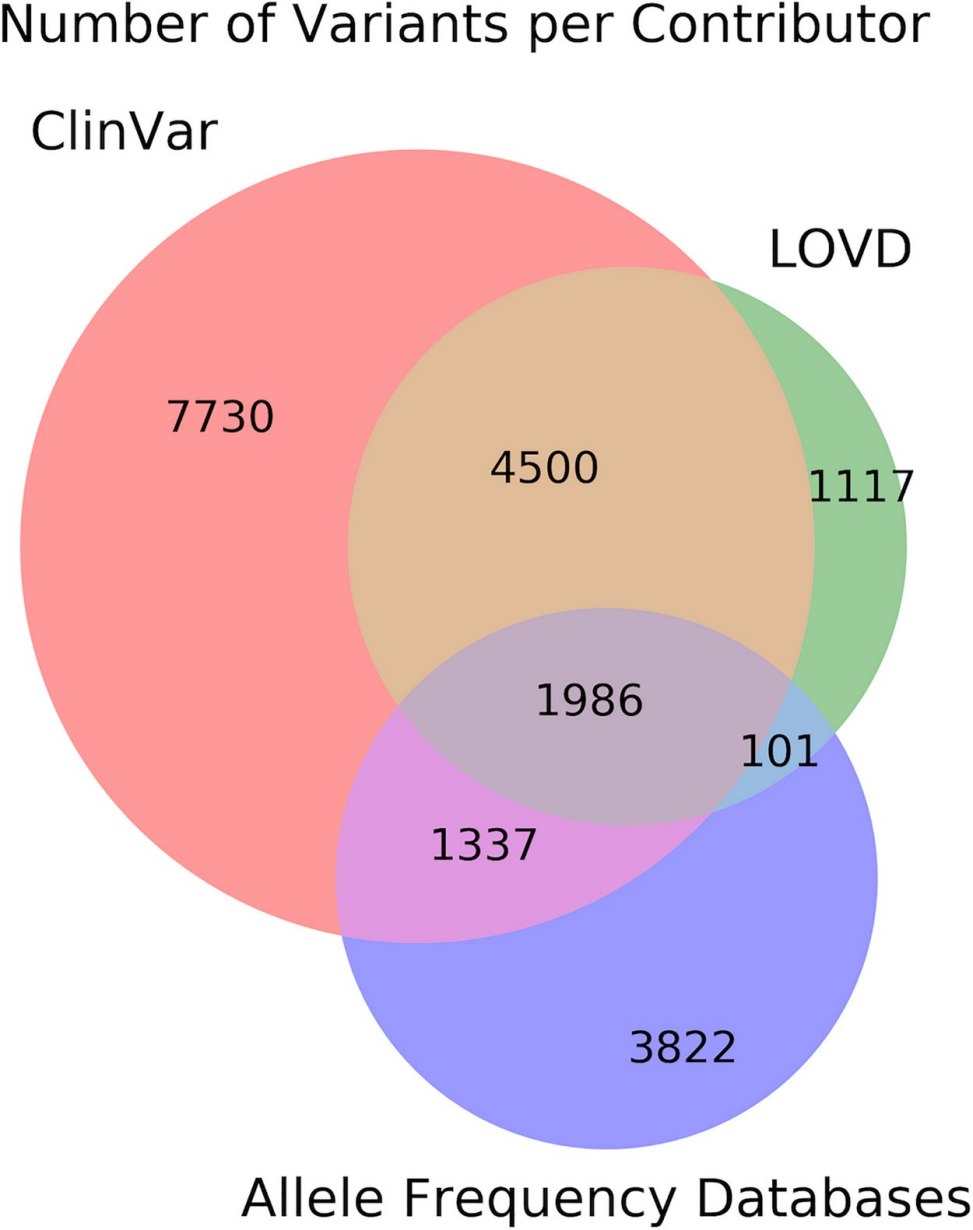
Venn diagram showing variants per large contributing data repositories, with variant overlap between repositories indicated. Allele frequency databases currently include ExAC, 1000 Genomes, and ESP. ESP, Exome Sequencing Project; ExAC, Exome Aggregation Consortium; LOVD, Leiden Open Variation Database.

The *BRCA1* and *BRCA2* variant data set comprises data from participating groups and major academic data sources, including ClinVar (with diverse submitters such as academic groups, commercial and hospital laboratories, and consortia such as the Consortium of Investigators of Modifiers of BRCA1/2 [CIMBA] [28, 29]), BIC (also represented in ClinVar) [30], Exome Aggregation Consortium (ExAC)/Genome Aggregation Database (gnomAD) [16], the 1000 Genomes Project, [31] the *BRCA1* and *BRCA2* Ex-UV Database displaying classifications based on multifactorial likelihood analysis [32], LOVD [33], NHLBI GO Exome Sequencing Project (ESP) [34], and the ENIGMA consortium [5]. Currently, the BRCA Exchange displays over 20,000 unique *BRCA1* and *BRCA2* variants; more than 6,100 of those variants are displayed with expert classifications from the ENIGMA Consortiumand to date approximately 3,700 have been deemed pathogenic. Variant numbers currently contributed by ClinVar, LOVD, and the three population frequency databases (ExAC, 1000 Genomes, and ESP) are shown in Fig 2, with unique and overlapping variants across those data repositories also indicated. Currently, the BRCA Exchange is the most comprehensive, publicly available representation of *BRCA1* and *BRCA2* variant information.

Ongoing work is focused on integration of new data and further annotation of the large set of VUSs; it is estimated that perhaps 80% to 85% of VUSs will ultimately be judged as nonpathogenic [4, 16], but large-scale aggregation of data is required to support this assertion [3–5, 17, 35]. To ensure the fidelity of the aggregated data, we filter the input data to ensure that the variants are consistent with the reference genome and are expressed in correct HGVS nomenclature. We address duplication in the genomic data by identifying and merging variants that produce equivalent allele strings. In some cases, we receive data on the same variants from multiple sources. For example, we receive variant classification data directly from ENIGMA and BIC and indirectly from these sources via ClinVar. Although these submissions may represent the same variants, they do not provide exactly the same information for each variant, and we adapt for this with separate tiles to show the variant attributes specific to each data source.

We have not yet addressed the issue of noise in the clinical phenotype or other sequence-unrelated data. One reason is because we intend to show data “as is” from various sources. To reduce the impact of this noise, the ENIGMA-curated classifications include high-level statements to support the assertions of the classifications. Moving forward, as we begin collecting individual-level data (see Box 2), it may be possible to assign value judgment on data depending on the source (e.g., recording discordances with expert-curated variants). Although it will likely be impractical and unethical to review actual clinical records for the most part, as an example, we may ask submitters for generic details on the source of their information and note these accordingly (e.g., self-reported cancer, cancer registry confirmed cancer, and cancer diagnosis extracted from pathology report provided to submitter).

**Box 2. Future plans for BRCA Challenge**

**Toward a comprehensive annotation of genetic variants of *BRCA1* and *BRCA2***

1. Establish consistency and collaboration in classifications across testing organizations and local databases
2. Consistent information for BRCA families internationally
3. Global representation of data deposition
4. Use collated individual-level data for developing new classification algorithms
5. Develop safe display solutions for case-level data

**Advances for technical development**

1. Define a standardized model for international data sharing
2. Develop APIs for distinct shareholders—both internationally and with respect to laboratory and healthcare providers
3. Pseudonymization software to accelerate annotation of remaining VUS

**Outreach**

1. Educate country-specific funding bodies on the need to develop a model for federated funding of data-sharing initiatives
2. Full engagement of patient advocacy

We are also exploring ways to avoid duplication of individual-level data because we plan to move to collection of such data. It is likely that ethical constraints regarding access to identifying information will require specific ethically approved protocols to allow curator access to identifying information in a secure environment that will allow for crosschecks, such as similarity in pedigree structure, ages, and types of cancer diagnoses. Access to additional accompanying genetic data—in particular, common variation—has utility to detect duplicated or closely related individuals. Complex pseudoanonymization protocols could prove useful to access deidentified data with need for individual-level consent (see below), but the issues around detecting and adjusting for potentially duplicated data points will remain.

In addition to the aggregation of large variant repositories, the BRCA Exchange works with data sets specific to local populations around the world, especially national initiatives such as the Brazilian Initiative on Precision Medicine (BIPMed), to support data submission. All national governments have been approached to encourage participation in the BRCA Challenge by the United Nations Education, Science and Culture Organization (UNESCO), coordinated by Global Variome (www.humanvariomeproject.org)—an associated nongovernmental organization, a member of GA4GH, and a partial sponsor of the BRCA Challenge. When possible, it is recommended that these projects structure data elements for inclusion using the LOVD open-source software or by direct upload to ClinVar, but it is also possible for these variant sources to add additional data elements to the Exchange directly in order to capture the most exhaustive evidence available.

The BRCA Exchange has initiated engagement with patient and advocacy groups, primarily focusing on getting input from key patient advocates and stakeholders with plans to raise awareness of the importance of data sharing among global patient and advocate communities and to encourage patient and advocate groups to engage with data holders to promote sharing of their data. A further goal is to develop tools to make the database meaningful and useful to patients and advocates, and to that end, a pilot effort recently released a mobile phone application for simple access to the variant database and push notifications when classifications are updated for variants of interest.

### Technical framework for BRCA Exchange

The BRCA Exchange software has been developed as open-source code found on https://github.com/BRCAChallenge/brca-exchange/. Technical development has been led by researchers at University of California, Santa Cruz, and ETH Zurich and hosted on the Microsoft Azure cloud. The central component of the BRCA Exchange software is the data analysis pipeline (Fig 1). Once per month, the automated pipeline downloads variant data from the ClinVar [36] and LOVD [33] variation repositories, the BIC [30] and BRCA Ex-UV [32] locus-specific repositories, and the ExAC [37], ESP [34], and 1000 Genomes [31] population genetics repositories. Additionally, the ENIGMA consortium [5] sends new variant interpretations to BRCA Exchange when submitted to ClinVar.

After collecting these data, BRCA Exchange determines the GRCh38 (Genome Reference Consortium Human Build 38 Organism) genomic coordinates of each variant with the HGVS python library [38] and CrossMap [39]. The pipeline identifies and removes variants with erroneous genomic representations, such as HGVS strings that are inconsistent with the HGVS standard and cannot be parsed, or variants for which the reference allele is no longer consistent with the reference genome. In the most recent release (version 21; July 2018), the pipeline detected and removed 1,330 erroneous variants. The pipeline then identifies and merges variants that are genomically equivalent by deriving an alternative allele string for each variant that consists of the variant plus flanking genomic bases, comparing these allele strings and merging any variants that yield equivalent allele strings. With this strategy, the pipeline detects equivalent variants that show no apparent similarity in their HGVS strings or genomic coordinates, such as complex indels that produce the same alternative allele string through differing combinations of insertions and deletions. Based on this strategy, sets of variants from the input repositories are merged into one set of distinct variants. In the most recent release, the pipeline merged 1,773 pairs of equivalent variants. BRCA Exchange publishes a Docker container on Dockerhub (https://dockerhub.com) to repeat these processing steps.

The pipeline also computes in silico predictions for each variant to indicate whether the variant is likely to disrupt splicing, introduce a premature stop codon, and/or impact a key domain or functional residue (see annotation below). These data are stored in a Postgresql database, in which they serve the BRCA Exchange web portal, mobile application, and application programming interface (API).

As part of this monthly update, BRCA Exchange tracks the update history of each variant. The update history is displayed in the variant details pages and includes dates of variant data updates, indications of the specific data updated, and links to download previous versions of the variant data. In addition to summarizing the changes for each variant, BRCA Exchange tracks and displays changes to ClinVar and LOVD for each variant to indicate how and when a clinical or functional interpretation has been updated by its submitter. Users can access versions of the variant data from any past date, in line with regulations for clinical-grade databases.

The variant data can be downloaded via the API or web portal. Under the data use policy, these data can be freely queried, downloaded, reused, and redistributed. The web portal offers downloads of the latest variant data and all previous data releases since the launch of the project. Each data release is distributed as a tarball that contains not just the final variant data but also the intermediate output files and log files that were generated by the pipeline execution, to increase the transparency and repeatability of these analysis steps. By using the BRCA Exchange Docker container published at Dockerhub, a user can replicate the variant merging and validation portions of the pipeline from this previous data release data for all releases subsequent to February 2018.

To assess the quality of the resulting data, we have compared the expert interpretations from ENIGMA and consensus classification based on available ClinVar, LOVD, BIC, and ENIGMA classifications with the variant interpretations from BRCA Share (S1 Table) [40]. BRCA Share is an independent repository of variants from the Universal Mutation Database (UMD) and the testing labs Quest Diagnostics and Laboratory Corporation of America (LabCorp). The contents of BRCA Share are available to academic researchers free of charge and to commercial partners under a subscription fee. We have not yet been able to integrate BRCA Share with the BRCA Exchange variants due to these differences in the fee structure (the contents of BRCA Exchange are freely available to all) but we would welcome the opportunity to do so in the future should this issue be surmounted. As revealed in the comparison, the repositories are largely concordant (S1 Table). Because the content of these repositories was developed independently, this concordance reflects favorably on the quality of the data. Each repository contains some variants that are not seen in the other, and in many cases, offers clinical interpretations of these variants. This point underscores the value of data sharing, and the potential for additional data sources to provide new knowledge.

### Annotation framework: Variant curation and interpretation

**Fig 3:**
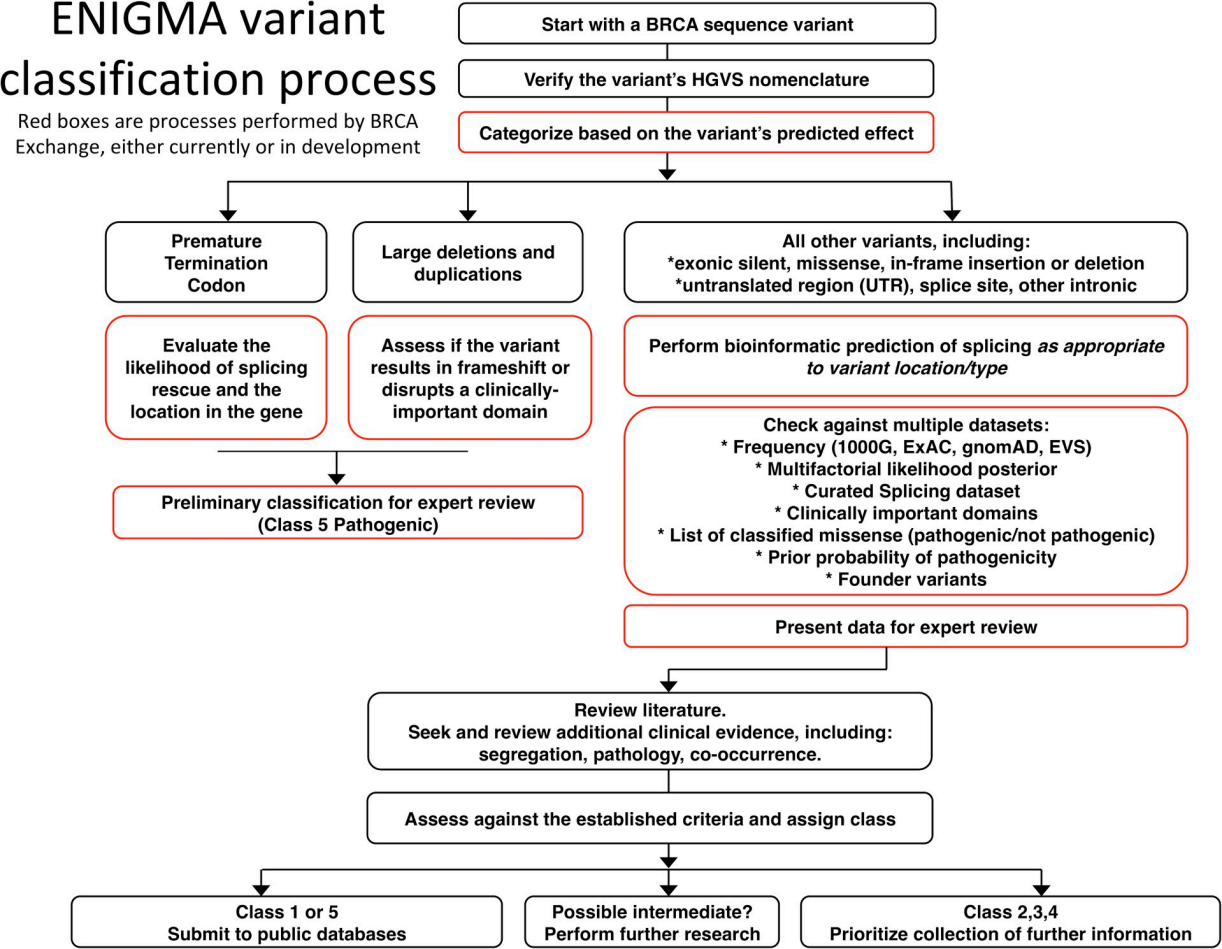
ENIGMA variant classification process. ENIGMA, Evidence-based Network for the Interpretation of Germline Mutant Alleles; ExAC, Exome Aggregation Consortium; gnomAD, Genome Aggregation Database; HGVS, Human Genome Variation Society; ins-del, insertion-deletions; UTR, untranslated region; 1000G, 1,000 Genomes Project.

The BRCA Exchange seeks to comprehensively gather or compute data for authoritative variant curation using classification approaches developed by the ENIGMA consortium and to display these classifications with supporting rationale. The detailed criteria developed by ENIGMA for *BRCA1* and *BRCA2* variant classification have been informed by clinically directed research and are publicly available (http://www.enigmaconsortium.org). These criteria will evolve as new information, including findings from ongoing research, accrues. The BRCA Exchange resource will incorporate data from many sources, including next generation sequence data from diagnostic laboratories, and data sets relevant for the ENIGMA variant classification process that have been collated from publicly available and /or other unpublished information and curated for accuracy. The key differences of the ENIGMA *BRCA1* and *BRCA2* classification criteria to generic classification criteria include the following: both qualitative and quantitative (statistically derived) approaches can be applied; variant interpretation considers a catalog of clinically important protein domains, derived by overlaying extensive knowledge of protein function and clinical effect of missense alterations; knowledge of naturally occurring mRNA transcripts has been used to inform the interpretation of possible spliceogenic variants; bioinformatic prediction of variant pathogenicity due to alteration at the level of protein (missense or in-frame deletion) or mRNA transcription (donor, acceptor, other exonic/intronic sequences like splice enhancers/silencers, and exonic de novo donor site) have been clinically calibrated for *BRCA1* and *BRCA2* sequences [16, 32] (Fig 3). It is notable that reference to population data sets is a key initial step used to parse variants classified as benign or likely benign because of frequency incompatible with high risk.

Automated processes to prioritize variants within the BRCA Exchange for streamlined classification and to support the continued curation of variants are under active development, including calculation of missense alteration prediction scores and prior probability of pathogenic as well as reference to knowledge of clinically important functional domains. In addition, variant annotation has been expanded to include automated calculation of splicing prediction scores and secondary annotation of predicted effects on mRNA splicing. This will include reference to basic variant position and/or effect annotation to define if a variant leads to gain or loss of splicing function, assessment of the score relationship to nearest donor or acceptor, as well as the predicted effect of likely mRNA aberration, e.g., out-of-frame versus in-frame. These will be made available via the BRCA Exchange and integrated into future versions of the automated variant annotation process.

### Ethical and regulatory considerations

In collaboration with the GA4GH Regulatory and Ethics Work Stream, the BRCA Exchange has considered the importance of local as well as global issues surrounding patient data sharing. Recognizing that barriers to data sharing are more than technical, the BRCA Challenge not only explores technical solutions such as container methods but also receives support and insight from a highly active Regulatory and Ethics Foundational Work Stream as part of its relationship as a Driver Project of GA4GH. At the same time, the national context can inform the use of data and protections required according to local laws and standards, adding complexity to the BRCA Exchange’s goal to aggregate diverse multinational resources. However, it is possible to develop shared solutions to enable annotated data to be shared widely and better inform the public as to the significance of specific variants in *BRCA1* and *BRCA2*—some of which could have widely differing allele frequencies based on population histories.

Sharing can result in extensive benefits through improved clinical care for patients, but it also brings concerns that public data may be misinterpreted by patients or providers or misused to the detriment of patients [41]. The responsibility to ensure data quality and responsible use within an online, large-scale data-sharing and curation community such as the BRCA Challenge needs to be carefully laid out, delegated, and clarified through data submission and data access and use agreements; for BRCA Exchange, these have been drafted with support from GA4GH to ensure maximal uptake from global contributors. BRCA Exchange is also preparing materials for genetic counselors as well as nonspecialist physicians and advocacy and support organizations, as well as considering engagement strategies with such organizations to enable their participation in design, governance, and oversight. To this end, engagement with patient advocacy groups has been a central goal—to ensure the community has an accurate and useful understanding of the public resources. All data were obtained from third-party sources and no additional ethics approval was required.

As the call for global data sharing increases, it is critical for the BRCA Challenge to develop strategies to enhance the ability to share variant annotation—and eventually primary data—through safe and protected mechanisms. One such mechanism is a container software architecture, in which data can be queried in its home institution, with anonymized and aggregated results exported. Additionally, the BRCA Challenge supports the use of pseudonymization software to enable a more comprehensive understanding the clinical significance of genetic variation in *BRCA1* and *BRCA2*, which, in turn, should lead to reclassification of a fraction of the current VUSs based on new informative data drawn from disparate sources, including diagnostic, clinical, and national nodes.

For example, a BRCA Challenge case-level national study is underway involving the National Cancer Registration and Analysis Service (NCRAS) in Public Health England that assembles, in near real time, the world’s most detailed clinical data set on all 530,000 cancers diagnosed by the National Health Service (NHS) each year in the 56 million people of England and Wales. To date, 14 of the country’s 17 genomic laboratories responsible for BRCA testing have fully engaged and have uploaded *BRCA1* and *BRCA2* variant data from over 30,000 patients using in-house open-source pseudonymization software; the irreversible pseudonymized reports are matched using NHS Number plus fuzzy matching based on other identifiers. The pseudonym allows anonymous records derived from the same individual to be linked without the identity of the individual being revealed. Individual clinical data is also encrypted with its own key and only matching patients' clinical data is decrypted and made available to the NCRAS's cancer registration database. Data is collected by the NCRAS under Regulations 2 and 5 of Section 251 of the NHS Act 2006. The Regulation provides the legal gateway to set aside the common-law duty of confidence to allow data to be collected without direct consent to support healthcare functions. This is validated by the local “Caldicott” data guardian at each center. This approach pioneers the hypothesis-free linkage of molecular variants with electronic patient records to build an unbiased prospective knowledge base of cancer predisposition.

### Future directions

The BRCA Challenge represents an important step in the sharing and collaborative annotation of *BRCA1* and *BRCA2* variants to advance human health globally. The urgent need to develop stable and comprehensive solutions for sharing data in cancer predisposition and other disease syndromes has been articulated by many, especially in response to the avalanche of clinical testing underway across the globe [42–45]. Because much of this is not necessarily driven by the classical paradigms based on familial risk, the BRCA Challenge is positioned to provide a stable, global solution that accounts for important differences with respect to academic, commercial, and national interests through the development of tools such as the BRCA Exchange and its corresponding mobile application. The BRCA Challenge is positioned to bridge gaps between academic approaches to variant classification—often conducted in familial collections—with population-based next-generation sequence data. The inclusion of this latter data source has begun to alter how and in what context risk for cancer could be estimated.

For the BRCA Challenge, the first development phase of a mobile application—as part of the BRCA Exchange—is complete, allowing individuals to track altered classification of variants. In turn, this information should be useful for clinicians and patients faced with decisions that will continue to be driven by recommending bodies and the critical relationship between patient and care provider. Engagement with patient advocacy groups as well as national programs should provide guidance to refine mobile applications to optimally fit the needs of many different constituents worldwide. The commitment of the BRCA Challenge to global data sharing—both for *BRCA1* and *BRCA2* variants as well as creating a standard applicable to other genes—can only succeed with involvement of the full spectrum of stakeholders.

As the project progresses, next steps will include collaboration in data aggregation and interpretation with more global data generators and data holders, continued technical development, and increased engagement with patients and patient advocates from around the world to further this important work (Box 2). With the development of the BRCA Exchange, our dedicated team has delivered a solution to improve data sharing and variant classification in the *BRCA1* and *BRCA2* genes and thus to improve clinical care for clinicians, patients, and their families. The BRCA Challenge serves as a demonstration project to bring together clinicians, researchers, data scientists, patients, and advocates to work toward a new model of data sharing. Its purpose is to create a comprehensive data-sharing and curation system that could be easily applied to the more than 114 other cancer predisposition genes identified to date [46], and ultimately, to all genes underlying recognized disease phenotypes, currently estimated to be around 3,000. In conclusion, the BRCA Challenge is a model for systematically engaging broad communities and resources for sharing data across genes and eventually regulatory regions of major clinical importance.

## Supporting information

S1 Table Comparison of BRCA Exchange to BRCA Share. The BRCA Share [40] repository contains variants from the UMD and the testing labs Quest Diagnostics and Laboratory Corporation of America (LabCorp). The contents of BRCA Share are available to academic researchers free of charge and to commercial partners under a subscription fee. To assess ENIGMA’s concordance with BRCA Share, we analyzed 5,452 variants—most of the BRCA Share set—by running them through the BRCA Exchange pipeline. BRCA Share contained 3,938 variants not assessed by ENIGMA, and BRCA Exchange contained 4,640 variants that were assessed by ENIGMA but not found in BRCA Share. In addition, we considered a consensus classification based on available ClinVar, LOVD, BIC, and ENIGMA classifications. For these consensus classifications, there were 2,228 variants in BRCA Share with no reference assertion in BRCA Exchange and 13,341 with reference assertions in BRCA Exchange but no assessment in BRCA Share. We compared the clinical significance assessments of the BRCA Share variants with the 6,154 assessments from ENIGMA and the 16,565 BRCA Exchange consensus reference interpretations. Overall, the clinical assessments showed a strong similarity. “Most Neutral” or “Likely Neutral” variants in BRCA Share were assessed as “Benign” or “Likely Benign” in BRCA Exchange, and most “Causal” or “Likely Causal” variants in BRCA Share were assessed as “Pathogenic” or “Likely Pathogenic” in BRCA Exchange. The largest differences were in the VUS counts. BRCA Share contained 310 variants designated as VUS that were assessed by ENIGMA—296 as “Benign” or “Likely Benign” and 14 as “Pathogenic.” BRCA Share also contained 473 variants designated VUS that were assessed in BRCA Exchange consensus—431 as “Benign” or “Likely Benign,” and 42 as “Pathogenic” or “Likely Pathogenic” (there are also 1,177 variants listed as VUS in both BRCA Share and BRCA Exchange consensus). In comparison to the number of variant classifications analyzed (5,552 from BRCA Share, 6,154 from ENIGMA, 16,565 from BRCA Exchange), this represents a high rate of concordance, which speaks favorably to the overall quality of the public variation data. Furthermore, the amount of new variant information in BRCA Share, beyond that which is available in BRCA Exchange, illustrates the potential gains of expanding data-sharing efforts. BIC, Breast Cancer Information Core; ENIGMA, Evidence-based Network for the Interpretation of Germline Mutant Alleles; LOVD, Leiden Open Variation Database; UMD, Universal Mutation Database; VUS, variants of uncertain significance.
(XLSX)
